# Tree rows in temperate agroforestry croplands alter the composition of soil bacterial communities

**DOI:** 10.1371/journal.pone.0246919

**Published:** 2021-02-10

**Authors:** Lukas Beule, Petr Karlovsky

**Affiliations:** Molecular Phytopathology and Mycotoxin Research, Faculty of Agricultural Sciences, University of Goettingen, Goettingen, Germany; Centro de Edafologia y Biologia Aplicada del Segura, SPAIN

## Abstract

**Background:**

Tree-based intercropping (agroforestry) has been advocated to reduce adverse environmental impacts of conventional arable cropping. Modern agroforestry systems in the temperate zone are alley-cropping systems that combine rows of fast-growing trees with rows of arable crops. Soil microbial communities in these systems have been investigated intensively; however, molecular studies with high taxonomical resolution are scarce.

**Methods:**

Here, we assessed the effect of temperate agroforestry on the abundance, diversity and composition of soil bacterial communities at three paired poplar-based alley cropping and conventional monoculture cropland systems using real-time PCR and Illumina sequencing of bacterial 16S rRNA genes. Two of the three systems grew summer barley (*Hordeum vulgare*); one system grew maize (*Zea mays*) in the sampling year. To capture the spatial heterogeneity induced by the tree rows, soil samples in the agroforestry systems were collected along transects spanning from the centre of the tree rows to the centre of the agroforestry crop rows.

**Results:**

Tree rows of temperate agroforestry systems increased the abundance of soil bacteria while their alpha diversity remained largely unaffected. The composition of the bacterial communities in tree rows differed from those in arable land (crop rows of the agroforestry systems and conventional monoculture croplands). Several bacterial groups in soil showed strong association with either tree rows or arable land, revealing that the introduction of trees into arable land through agroforestry is accompanied by the introduction of a tree row-associated microbiome.

**Conclusion:**

The presence of tree row-associated bacteria in agroforestry increases the overall microbial diversity of the system. We speculate that the increase in biodiversity is accompanied by functional diversification. Differences in plant-derived nutrients (root exudates and tree litter) and management practices (fertilization and tillage) likely account for the differences between bacterial communities of tree rows and arable land in agroforestry systems.

## Introduction

Modern temperate agroforestry systems are monoculture alley-cropping systems where rows of fast-growing trees (e.g. poplar (*Populus*) species) are alternated with rows of annual crops (tree-based intercropping). The cultivation of trees and crops in close spatial proximity allows various interspecific interactions that can result in complementary use of the resources [1]. Partition of resources between trees and crops in agroforestry systems is regarded as the key advantage of agroforestry over conventional arable croplands [2]. Agroforestry systems can reduce nitrate leaching through nitrate uptake by deep-rooting tree roots expanding below the cropland zone (‘safety-net’ role of tree roots) [3–5] and increase soil fertility through tree-litter input [6, 7]. Furthermore, agroforestry can increase faunal and floral diversity compared to conventional monoculture cropland, as reviewed by Udawatta *et al*. [8]. Temperate agroforestry consistently diminished crop yield close to the trees [9–11], yet maintained food safety requirements for small-grain cereals [12]. The benefits of integrating trees in agricultural systems have been recognized and improvement of the sustainability of agriculture through agroforestry has been proposed [13, 14].

Over the last two decades, soil microbial communities in temperate agroforestry systems have been extensively investigated using traditional methods such as fumigation-extraction, enzyme activities, and substrate-induced respiration. Soil microbial biomass has been shown to be greater in agroforestry than in conventional monoculture cropland [15, 16], whereas in poplar-based agroforestry, the increase in microbial biomass was limited to the tree rows [17]. Similarly, the bacterial biomass in an adler (*Alnus rubra*)-maize (*Zea mays*) agroforestry system was greater in the vicinity of the tree rows [18]. Furthermore, numerous studies of soil microbial communities in temperate agroforestry systems found more diverse catabolic potential [19–23] and greater efficiency of substrate use under agroforestry than in conventional cropland [17, 24]. It is reasonable to assume that these differences resulted from compositional differences among soil microbial communities. In 2013, Bardhan and co-workers [25] tested this hypothesis by investigating the composition and diversity of soil bacterial communities in a maple (*Acer saccharinum*)-based agroforestry system using denaturing gradient gel electrophoresis (DGGE). The authors did not detect any difference between the composition or diversity of soil bacteria in the tree and crop rows, which was likely due to limited coverage of complex communities by DGGE [26]. Despite the wide application of next generation sequencing (NGS) techniques in soil microbiology, sequencing of amplified segments of ribosomal RNA genes or spacers, which provides a high sampling depth and taxonomical resolution for cultivable and non-cultivable microorganism, has rarely been used in studies of agroforestry. Recently, Banerjee *et al*. [27] investigated bacterial communities in Canadian agroforestry systems using both quantitative (real-time PCR (qPCR)) and qualitative (amplicon sequencing) molecular approaches. The authors concluded that temperate agroforestry did not promote soil bacterial diversity but it increased bacterial abundance [27]. Likewise, a recent molecular investigation revealed that tree rows of agroforestry systems promoted the abundance of several bacterial groups in soil [28]. Additionally, temperate agroforestry has been shown to alter the abundance of microorganisms involved in N cycling [28, 29]. Despite these recent insights, systematic investigations of soil bacterial communities in temperate agroforestry systems with high taxonomical resolution are missing.

The present study aimed to assess the effect of poplar-based temperate alley cropping (agroforestry) on bacterial communities in soil. We expected that differences in the distribution of tree litter input [11] as well as the fertilization and tillage regime [30] between agroforestry tree and crop rows and monoculture croplands would result in changes of the soil microbiome as reported previously [27–29]. Therefore, we hypothesized that the integration of rows of trees into arable land through agroforestry i) increased the abundance and ii) diversity of soil bacteria and iii) affected the composition of the soil bacterial community along transects from the centres of the tree rows to the centres of the agroforestry crop rows. Furthermore, this study aimed to establish a novel technique for library normalization using qPCR and gel densitometry.

## Materials and methods

### Study sites and experimental design

**Fig 1:**
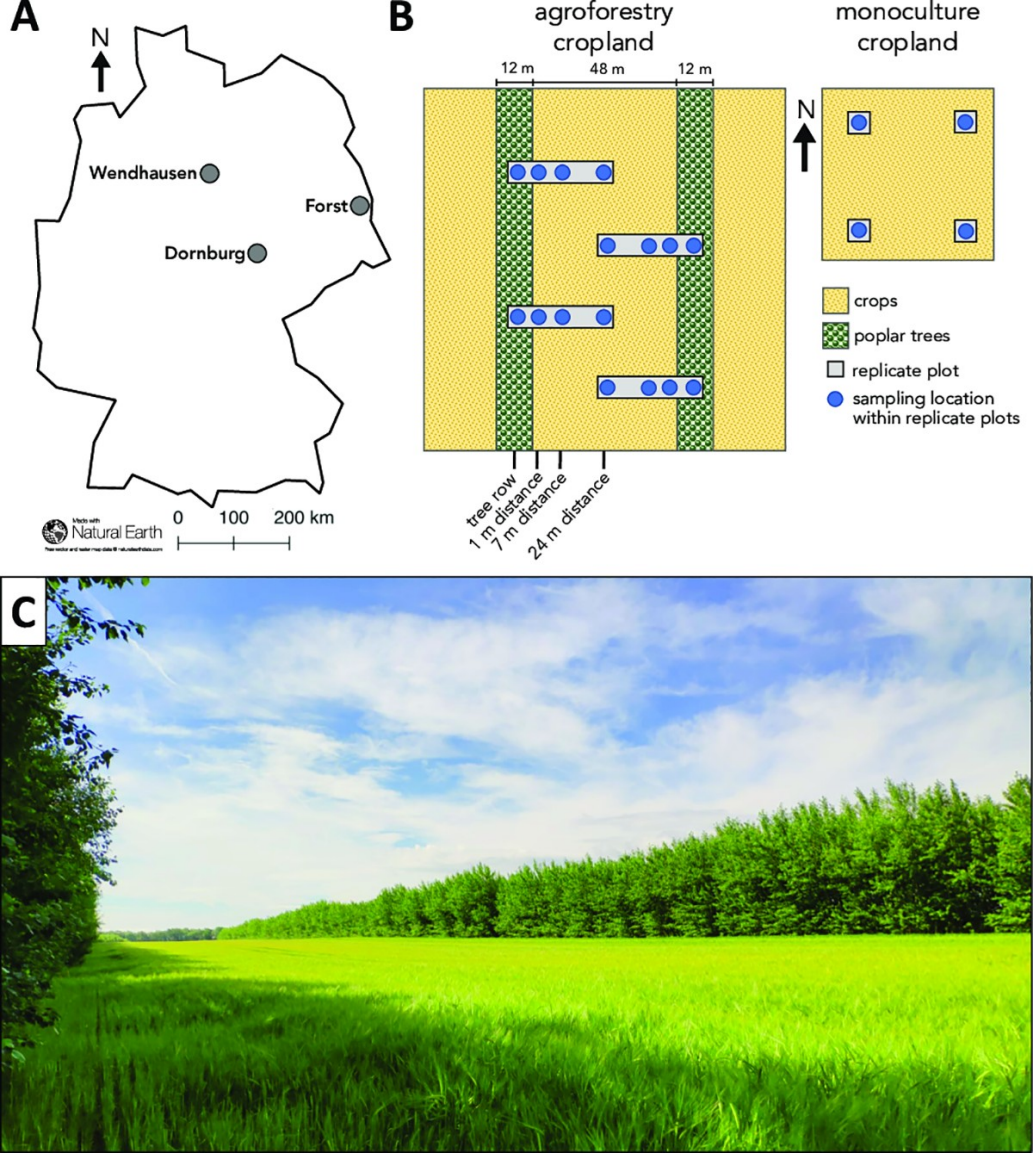
Study sites in Germany (**A**), experimental design of paired agroforestry and conventional monoculture cropland (**B**), and a picture taken at the tree-crop interface of the agroforestry cropland system at Dornburg (**C**). The soil types at the three study sites were Calcaric Phaeozem soil (near Dornburg, Thuringia), Gleyic Cambisol soil (near Forst, Brandenburg), and Vertic Cambisol soil (near Wendhausen, Lower Saxony). Photo taken by G. Shao.

The land owners gave their permission to conduct soil sampling on their property. We chose three study sites with paired agroforestry and conventional monoculture cropland in Germany (Fig 1). The soil types at the three study sites were Calcaric Phaeozem soil (near Dornburg, Thuringia), Gleyic Cambisol soil (near Forst, Brandenburg), and Vertic Cambisol soil (near Wendhausen, Lower Saxony) (Table 1). The agroforestry systems were established between 2007 and 2010 by converting conventional monoculture cropland into an alley-cropping system. At every site, 12-m wide rows of poplar trees were planted from cuttings (clone Max1; *Populus nigra* × *P*. *maximowiczii*) using a dibble bar. The tree rows were North-South oriented and interspersed with 48-m wide rows of crops (Fig 1B). The agroforestry crop rows were managed identically as the monoculture croplands on the same sites (identical crop rotation, fertilization, and pesticide treatment). The crop rotations included maize (*Zea mays*), summer and winter barley (*Hordeum vulgare*), winter oilseed rape (*Brassica napus*), and winter wheat (*Triticum aestivum*) (Table 1). Fertilizer was applied to the crop rows of the agroforestry systems and the monoculture croplands in spring according to standard practice (Table 1). Following common temperate agroforestry practice, the trees rows of the agroforestry systems did not receive fertilizer [30].

**Table 1:** Site characteristics and management at the three study sites of paired temperate agroforestry and monoculture cropland.

study site	Dornburg	Forst	Wendhausen
**location**	51°00’40”N, 11°38’46”E	51°47’11”N, 14°38’05”E	52°20’00”N, 10°37’55”E
**soil type**	Calcaric Phaeozem	Gleyic Cambisol	Vertic Cambisol
**mean annual air temperature (1981–2010)**	9.9 ± 0.1°C^a^^,^^b^	9.6 ± 0.2°C^a^^,^^c^	9.6 ± 0.2°C^a^^,^^d^
**mean annual precipitation (1981–2010)**	608 ± 21 mm^a^^,^^b^	568 ± 21 mm^a^^,^^c^	637 ± 23 mm^a^^,^^d^
**meters above sea level**	289 m	67 m	82 m
**year of agroforestry system establishment**	2007	2010	2008
**harvest(s) of the aboveground tree biomass of the agroforestry system**	January 2015	February 2015, March 2018	January 2014
**crop rotation (2016–2017–2018–2019)**	summer barley–winter oilseed rape–winter wheat–summer barley	winter wheat–winter barley–maize–summer barley	winter oilseed rape–winter wheat–winter wheat–maize
**fertilization rates in 2019 (kg N–P–K ha**^**-1**^ **yr**^**-1**^**)**	36–22–31	42–8–27	101–0–0

^a^Mean ± standard error during 1981–2010.

^b^Climate station at Jena (station ID: 2444) of the German Meteorological Service.

^c^Climate station at Cottbus (station ID: 880) of the German Meteorological Service.

^d^Climate station at Braunschweig (station ID: 662) of the German Meteorological Service.

At every site, we established four replicate plots in both the agroforestry and the monoculture cropland systems (Fig 1B). The replicate plots in the agroforestry systems were linear transects orthogonal to the North-South orientation of the tree rows. These transects spanned from the centre of the 12-m wide tree row to the centre of the 48-m wide agroforestry crop row. Soil samples were collected along the transects within the centre of the tree row (approximately 1 m from the poplar trunk) as well as in the agroforestry crop row at 1 m, 7 m, and 24 m (centre of the agroforestry crop row) distance from the trees (Fig 1B) (see https://doi.org/10.20387/BONARES-4984-ZWYR for spatial references of the sampling locations). In the adjacent monoculture systems, soil samples were collected in the centre of the plots. In total, 20 soil samples were collected at each site.

### Soil collection and DNA extraction

In 2019, soil samples of the top 5-cm depth were collected on July 15 (Vertic Cambisol), July 16 (Calcaric Phaeozem), and August 6 (Gleyic Cambisol). At each of the 20 sampling locations, soil was collected using three 250 cm^3^ stainless steel cylinders (5 cm height) per site and immediately thoroughly homogenised in a sterile plastic bag. Still in the field, an aliquot of 20 g of fresh soil was transferred into a 15 mL Falcon tube (SARSTEDT, Nümbrecht, Germany) and frozen at -20°C. Upon arrival in the laboratory, soil samples were freeze-dried for 72 h and finely ground using a swing mill (Retsch MM400, Retsch, Haan, Germany). DNA was extracted from 50 mg soil using a cetyltrimethylammonium bromide-based (CTAB) protocol described previously by Brandfass & Karlovsky [31]. Briefly, soil was suspended in 1 mL CTAB with 1 μL proteinase K, incubated at 42°C and subsequently at 65°C, and 800 μL phenol were added. The mixture was shaken, centrifuged, and the supernatant was extracted with aliquots of chloroform/isoamyl alcohol twice. DNA was precipitated using PEG/NaCl and pelleted by centrifugation. The pellets were washed with EtOH twice, dried, and re-dissolved in 50 μL 1 × TE buffer (10 mM Tris, 1 mM ethylenediaminetetraacetic acid (EDTA), adjusted to pH 8.0 with HCl). Extracted DNA was visualized in 0.8% (w/v) agarose gels (in 1 × TAE buffer (40 mM Tris, 20 mM sodium acetate, 1 mM Na_2_EDTA, adjusted to pH 7.6)) stained with ethidium bromide. Gel electrophoresis was carried out at 4.6 V cm^-1^ for 60 min. Soil DNA extracts were stored at -20°C. The extracts were tested for the absence of PCR inhibitors as described previously [32].

### Determination of absolute abundance of bacteria

Prior to library preparation, soil bacteria in the 60 DNA extracts were quantified and normalized using qPCR. Amplifications were performed in triplicates in a CFX 384 Thermocycler (Biorad, Rüdigheim, Germany) in 384-well microplates. The reaction volume was 4 μL consisting of double-distilled water (ddH_2_O); buffer (10 mM Tris-HCl, 50 mM KCl, 2.0 mM MgCl_2_, pH 8.3 at 25°C); 200 μM of each deoxynucleoside triphosphate (Bioline, Luckenwalde, Germany); 0.4 μM of each primer (341F (5’-CCTACG GGNGGC WGCAG-3’)/785R (5’-GACTAC HVGGGT ATCTAA KCC-3’) [33], targeting the V3–V4 region of the bacterial 16S rRNA); 0.1 × SYBR Green I solution (Invitrogen, Karlsruhe, Germany)); 1 μg μL^-1^ bovine serum albumin; 0.03u μL^-1^ Hot Start *Taq* DNA Polymerase (New England Biolabs, Beverly, Massachusetts, USA) and 1 μL of template DNA (standards or 1:50 dilutions of the DNA extracts in 0.5 × TE) or 0.5 × TE for the negative control. Standards in 0.5 × TE were obtained from environmental soil DNA extracts and amplified in triplicates. Standard rows were obtained from 1:2 serial dilutions of 1 pg PCR product. The quantification cycle (C_q_) values of the standards that covered the range of the samples were plotted against their log-transformed starting quantity. Curve fitting was conducted to account for non-linear PCR efficiency over the range of the standard row. A third order polynomial function was fitted and the absolute abundance of bacteria in the extracts was determined.

### Library normalization

To achieve a narrow distribution of the titre of libraries for NGS, the concentration of bacterial 16S rRNA genes in sample extracts was determined by qPCR (see Determination of absolute abundance of bacteria) and the extracts were diluted in 0.5 × TE to achieve the same concentration of rRNA genes in all samples. The success of normalization was tested by qPCR. The normalization was regarded as successful when the difference among C_q_ for all sample pairs was smaller than one. The samples that did not meet this criterion were re-diluted until successful normalization was achieved.

A second normalization was carried out by diluting the libraries based on their DNA concentration determined by densitometry. A 2 μL aliquot of the each of the 60 libraries was diluted 1:10 in 0.5 × TE buffer and 4 μL of the dilutions were visualized on 1.7% (w/v) agarose gels in sets of 20 samples per gel, resulting in three gels for the 60 samples. A standard row of 11 standards was obtained from 3:1 serial dilutions of a library in 0.5 × TE buffer. The standards were loaded onto a 1.7% (w/v) agarose gel in duplicates. The densitometric quantification of library DNA was performed employing ImageJ version 1.52q [34]. A second order polynomial function was fitted to obtain a calibration curve (S1 Fig). The relative library yield of each of the 20 samples per gel was determined. All samples loaded onto one gel were normalized to the sample with the lowest library yield within the respective gel and pooled, resulting in one pooled sample per gel. To ensure successful normalization across gels, 4 μL aliquots of the three pooled samples (one from each gel) were loaded on onto a 1.7% (w/v) agarose gel in duplicates. The densitometric results were used to normalize the pooled samples as described above for individual libraries.

### Library preparation and high-throughput Illumina sequencing

Amplifications for library preparation were carried out in a peqSTAR 96 universal gradient thermocycler (PEQLAB, Erlangen, Germany) in 25 μL reaction volumes consisting of ddH_2_O; buffer (10 mM Tris-HCl, 50 mM KCl, 2.0 mM MgCl_2_, pH 8.3 at 25°C); 200 μM of each deoxynucleoside triphosphate (Bioline, Luckenwalde, Germany); 0.4 μM of each primer (341F/785R [33]); 1 μg μL^-1^ bovine serum albumin; 0.03u μL^-1^ Hot Start *Taq* DNA Polymerase (New England Biolabs, Beverly, Massachusetts, USA) and 6.25 μL of normalized template DNA or 0.5 × TE for the negative control. The primers were a set of 48 dual-indexed primer pairs that included 0–3 frameshifting bases (Ns) to improve Illumina base-calling followed by an 8-bp barcode at the 5’-end of each primer. Thermocycling conditions were as follows: initial denaturation (95°C for 2 min), 3 touch-up cycles of denaturation (95°C for 20 sec), annealing (50°C for 30 sec), and elongation (68°C for 30 sec), 25 cycles of denaturation (95°C for 20 sec), annealing (58°C for 30 sec), and elongation (68°C for 30 sec), and final elongation (68°C for 5 min). All libraries were prepared within one PCR run using the same mastermix. The libraries were visualized on 1.7% (w/v) agarose gels (in 1 × TAE buffer) stained with ethidium bromide. Gel electrophoresis was carried out at 4.6 V cm^-2^ for 60 min.

Based on the densitometry, the libraries were normalized (see Library normalization) and sent to LGC Genomics (Berlin, Germany) for adapter ligation using a commercial kit (Ovation^®^ Rapid DR Multiplex System 1–96 (NuGEN, San Carlos, CA, USA)). Finally, the libraries were sequenced in one multiplex sequencing run using the Illumina MiSeq Reagent Kit v3 (2 × 300 bp) (Illumina, San Diego, CA, USA) at the facilities of LGC Genomics, Berlin, Germany. Sequencing data have been deposited at NCBI’s Sequence Read Achieve (BioProject PRJNA667193).

### Processing of sequencing data

Raw paired-end data (10,736,108 reads in total) were demultiplexed using Illumina’s bcl2fast version 2.17.1.14 (Illumina, San Diego, CA, USA) and sorted by their barcodes (allowing 1 mismatch per barcode; missing, one-sided or conflicting barcode pairs were discarded). Sequencing adapters were clipped and reads shorter than 100 bp were discarded. Primer were clipped (allowing 3 mismatches per primer) and reads were imported in QIIME 2 version 2019.10 [35]. We employed DADA2 [36] for quality-filtering, merging, chimera and singleton filtering of the reads, and clustering of reads in exact amplicon sequence variants (ASVs) [37, 38]. The dereplicated ASVs were taxonomically assigned against the SILVA SSU database (release 132) [39] using VSEARCH [40]. Non-bacterial reads were removed from the obtained ASV table. After filtering, 5,890,832 bacterial counts were obtained. We normalized the library size to 12,521 counts per sample using scaling with ranked subsampling [41] using the ‘SRS’-function in the ‘SRS’ R-package version 0.1.0 [41] in the R environment version 3.6.1 [42]. Finally, our normalized dataset contained 40,708 ASVs.

### Quantification of *nifH* gene in soil

The most frequently used marker gene for N fixation by bacteria is *nifH*, which encodes for a subunit of the dinitrogenase reductase enzyme [43]. The abundance of *nifH* in soil was determined using qPCR as described previously. Briefly, qPCR reactions were performed in 4 μl reaction volume in 384-well microplates using a CFX384 Thermocycler (Bio-Rad, Rüdigheim, Germany). The reaction volume contained 3 μl mastermix and 1 μl of a 1:50 dilution in 0.5 × TE buffer of the DNA extracts or ddH_2_O for negative controls. The composition of the mastermix as well as primers and the thermocycling conditions have been described in details previously [28].

### Statistical analysis

Alpha diversity was quantified by calculating the Shannon index (*H’*) and the Pielou’s evenness index (*J’*) for the ASV count data. *H’* was determined using the ‘diversity’-function in the ‘vegan’ R-package version 2.5–6 [44]. *J’* was determined by dividing *H’* by the natural logarithm of the total number of species. The effect of sampling location (tree row, 1 m, 7 m, and 24 m distance from the tree row within the agroforestry crop row and monoculture cropland) within one soil type on alpha diversity measures, bacterial 16S rRNA and *nifH* gene abundance was determined using one-way analysis of variance (ANOVA) with Tukey’s honestly significant difference (HSD) test or Kruskal–Wallis test with multiple comparison extension.

The Bray-Curtis index of dissimilarity for pairwise comparisons was calculated from the square root-transformed ASV count data using the ‘vegdist’-function in the ‘vegan’ R-package version 2.5–6 [44]. The Bray-Curtis dissimilarities were visualized using non-metric multidimensional scaling (NMDS) with 100 random starts (‘metaMDS’-function in the ‘vegan’ R-package version 2.5–6 [44]). Additionally, we calculated the Bray-Curtis index of similarity (1 –Bray-Curtis dissimilarity) and visualized the similarity across samples using a network approach employing the ‘igraph’ R-package version 1.2.4.2 [45]. For the network visualization, edges were only drawn between samples with a Bray-Curtis index of similarity above the 75^th^ quantile. The network was spatially arranged applying the Fruchterman-Reingold algorithm [46]. The effect of soil type, sampling location, and soil type × sampling location on community composition was determined employing permutational multivariate analysis of variance (PERMANOVA) with 999 permutations using the ‘adonis2’-function in the ‘vegan’ R-package version 2.5–6 [44]). Complementary, we tested for the multivariate homogeneity of group dispersions (PERMDISP) on Bray-Curtis dissimilarities with 999 permutations (‘betadisper’-function in the ‘vegan’ R-package version 2.5–6 [44]).

The relative abundance of all identified genera was visualized and inspected. The effect of sampling locations within one soil type on the absolute abundance of bacterial 16S rRNA and *nifH* genes and the relative abundance of bacterial genera was determined using one-way ANOVA with Tukey’s HSD test or Kruskal–Wallis test with multiple comparison extension. One-way ANOVA with Tukey’s HSD was partly carried out on square root- or log-transformed data. For visualization purposes, the relative abundances of selected genera were Z-score normalized. Z-scores (Z_i_) for the relative abundance of each genus was calculated by subtracting the mean relative abundance of a genus (μ) from the relative abundance the genus in sample i (x_i_) and dividing the difference it by the standard deviation of the genus over all samples (σ):

$$Z_{i}=\frac{x_{i}-\mu }{\sigma }$$

Statistical analysis was performed in the R environment version 3.6.1 [42].

## Results

### Validation of two-step normalization of NGS libraries

**Fig 2:**
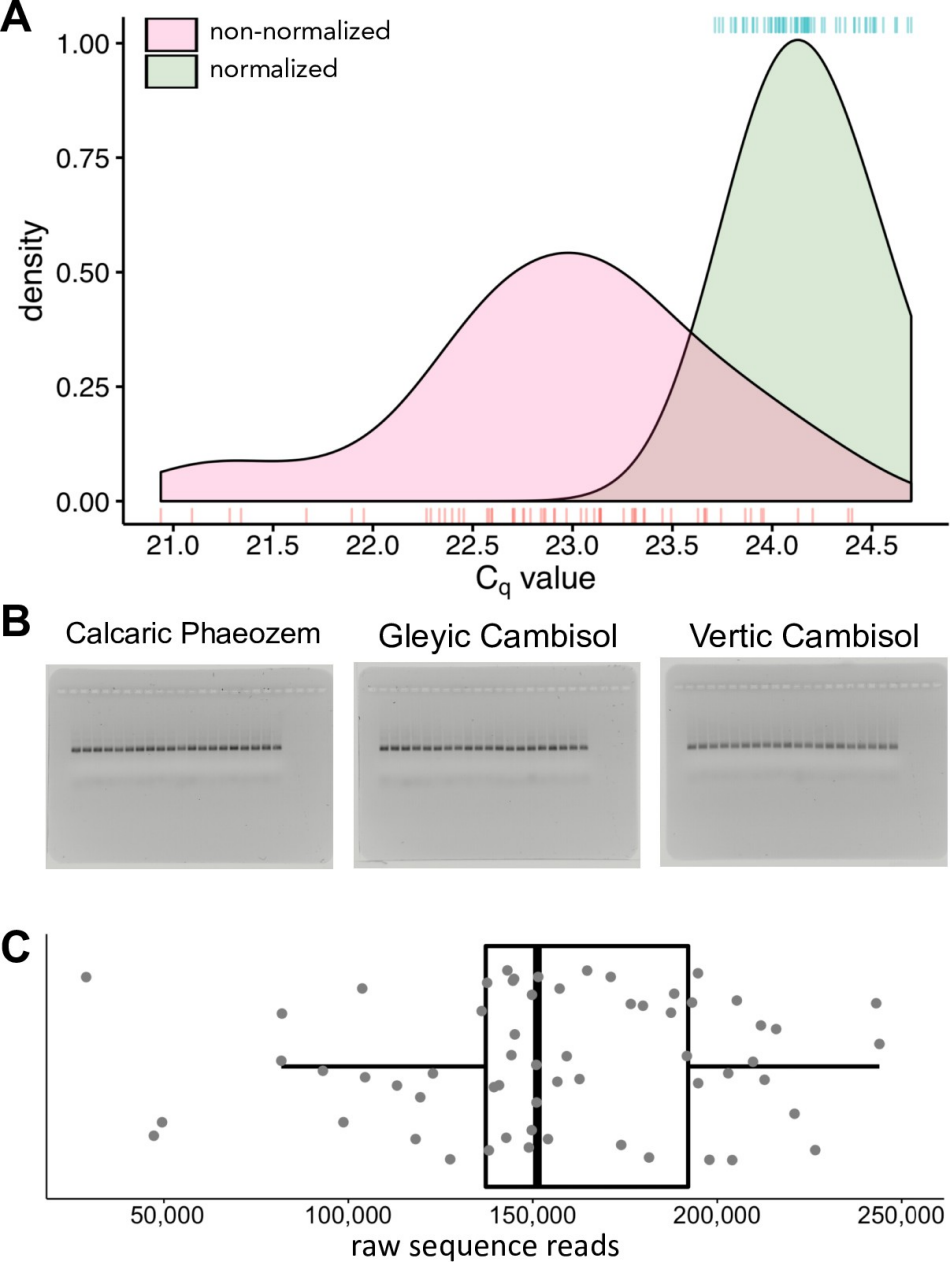
Kernel density estimation of the quantification cycle (C_q_) values for non-normalised and normalised samples (prior to library preparation) (**A**), prepared libraries normalized by photo densitometry (**B**), and the distribution of the raw sequence reads per library (**C**). Individual C_q_ values are plotted as marks below the density curves for non-normalised and above for normalised samples; curves were smoothened using a bandwidth of 0.3 (**A**). The box indicates the q25, q50, and q75, whiskers range from q25 or q75 to 1.5 × the interquartile range, dots represent individual data points (**C**). *n* = 60 samples.

Normalization of DNA samples prior to library preparation based on the quantification of bacterial 16S rRNA genes by qPCR substantially reduced differences in library size among samples (Fig 2A). To achieve a narrow distribution of C_q_ values for libraries, DNA extracts had to be diluted up to 7.5-fold. The libraries were further normalized by dilution up to 2.4-fold, based on the quantification of DNA concentration in the libraries by densitometry (Fig 2B). The number of raw sequence reads obtained from the normalized libraries ranged from 28,935 to 243,974 reads per library with an interquartile range from 137,234 to 192,129 (Fig 2C).

### Overall soil bacterial community

**Fig 3:**
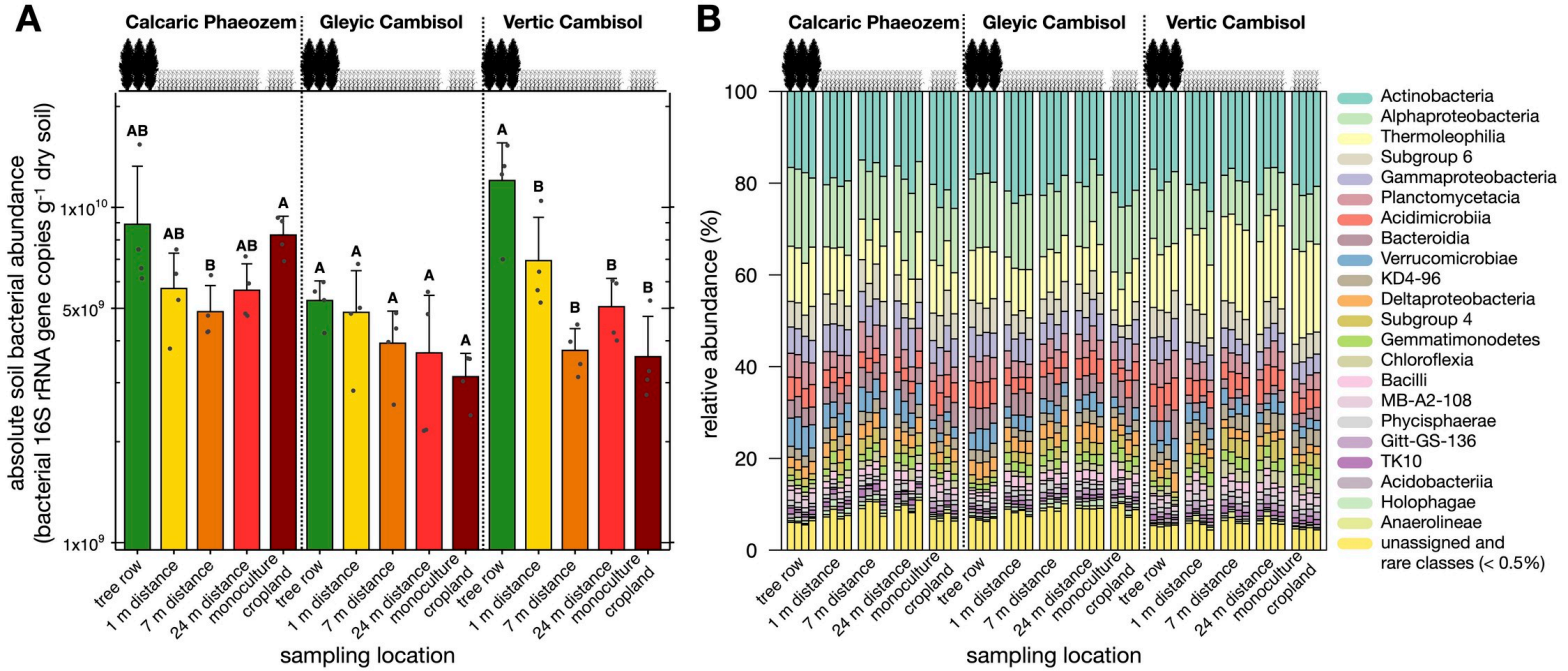
16S rRNA gene abundance of soil bacteria (**A**) and relative abundance of taxonomic groups of soil bacteria at class level (**B**) in paired temperate agroforestry and monoculture cropland systems in three different soil types. Samples in the agroforestry systems were collected in the tree row as well as at 1 m, 7 m, and 24 m distance from the tree row within the agroforestry crop row. Bars represent means with standard deviation (*n* = 4 per soil type × sampling location) (**A**). Different uppercase letters indicate significant differences among the sampling locations (one-way analysis of variance with Tukey’s honestly significant difference test or Kruskal–Wallis test with multiple comparison extension) (**A**). Soil bacterial classes with an overall relative abundance of < 0.5% were considered as rare classes and merged with unassigned classes (**B**).

The determination of the soil bacterial community size by qPCR revealed no differences among sampling location on the Calcaric Phaeozem and Gleyic Cambisol soil, except lower bacterial abundance on the Calcaric Phaeozem soil at 7 m distance from the tree row within the agroforestry crop row compared to the monoculture system (p = 0.05) (Fig 3A). On the Vertic Cambisol, soil bacteria were more abundant in the tree row than at all distances from the tree row within the crop row of the agroforestry system as well as in the monoculture cropland system (p ≤ 0.023) (Fig 3A).

The 40,708 ASVs found in the normalized bacterial reads were distributed among 10 dominant (≥ 0.5% overall relative abundance across all samples) and 28 rare phyla (< 0.5% overall relative abundance across all samples). Actinobacteria (38.8%) followed by Proteobacteria (21.8%), Acidobacteria (10.7%), Chloroflexi (9.0%), and Planctomycetes (5.5%) were the most abundant phyla. Overall, 99.8% of all taxonomic groups were assigned at phylum level. Alphaproteobacteria (13.9%), Actinobacteria (19.9%), Thermoleophilia (12.7%), Subgroup 6 (5.5%), and Gammaproteobacteria (5.3%) were the most abundant bacterial classes (Fig 3B). At genus level, *Nocardioides* (3.2%), *Microlunatus* (3.0%), and *Sphingomonas* spp. (2.9%) were most abundant across samples.

### Diversity and composition of soil bacterial communities

**Fig 4:**
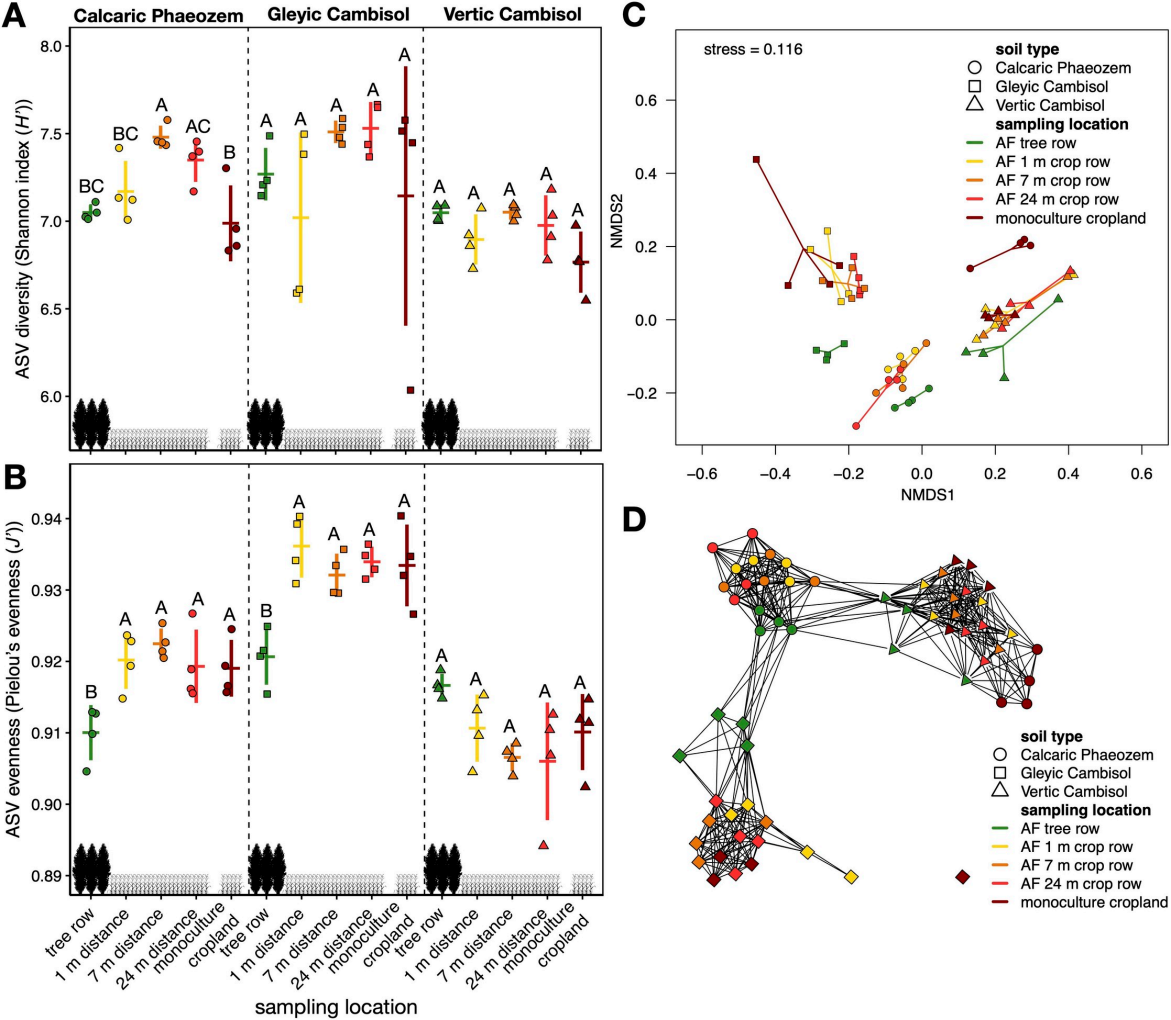
Alpha diversity measures (Shannon index (*H’*) (**A**) and Pielou’s evenness index (*J’*) (**B**)), non-metric multidimensional scaling (NMDS) of Bray-Curtis dissimilarities (**C**), and Bray-Curtis similarity network (**D**) of soil bacterial ASVs in paired temperate agroforestry and monoculture cropland systems in three different soil types. Samples in the agroforestry systems were collected in the tree row as well as at 1 m, 7 m, and 24 m distance from the tree row within the agroforestry crop row. Coloured vertical bars represent the standard deviation, coloured horizontal bars the mean (**A**, **B**). Coloured shapes represent individual data points (**A**, **B**). Different uppercase letters indicate statistically significant differences at p < 0.05 (one-way analysis of variance with Tukey’s honestly significant difference test or Kruskal–Wallis test with multiple comparison extension) (**A**, **B**). Solid lines in the NMDS span from the centroid of each group to the individual data points (**C**). Edges in the similarity network were drawn between samples with a Bray-Curtis similarity ≥ 0.75 (**D**). The network was spatially arranged applying the Fruchterman-Reingold algorithm (**D**). AF = agroforestry system, ASV = amplicon sequence variant.

Shannon diversity of ASVs on the Calcaric Phaeozem soil was greater in the agroforestry crop row at 7 m distance from the tree row than in the tree row, 1 m within the crop row and the monoculture system (p ≤ 0.048) (Fig 4A). On the Vertic Cambisol and Gleyic Cambisol soil, no differences in Shannon diversity among sampling locations were present. On the Calcaric Phaeozem and Gleyic Cambisol soil, Pielou’s evenness was lower in the tree row than in the agroforestry crop row and the monoculture (p ≤ 0.038) (Fig 4B). On the Vertic Cambisol soil, no differences in evenness were found among sampling locations.

The soil bacterial community composition was strongly influenced by soil types (PERMANOVA; *F* = 15.021, p = 0.001), which explained 28.8% of the observed variance (S1 Table). Furthermore, the interaction of soil type × sampling location explained 16.2% of the variance and was found to influence the community composition (PERMANOVA; *F* = 2.110, p = 0.001). The influence of sampling locations within each soil type on community composition was reflected by the large proportion of ASVs unique to the different sampling locations (S2 Fig). Furthermore, proportions of ASVs shared between tree rows and arable land (crop rows and monocultures) generally declined with distance from the tree rows (S2 Fig). Finally, the different sampling locations across soil types explained 12.0% of the variance and affected the composition of the bacterial community (PERMANOVA; *F* = 3.120, p = 0.001). Multivariate homogeneity of group dispersions was given for soil type, sampling location, and soil type × sampling location (PERMDISP; p ≥ 0.057). These results were reflected by the NMDS (Fig 4C) and the similarity network (Fig 4D).

### Relative abundance of taxonomic soil bacterial groups

**Fig 5:**
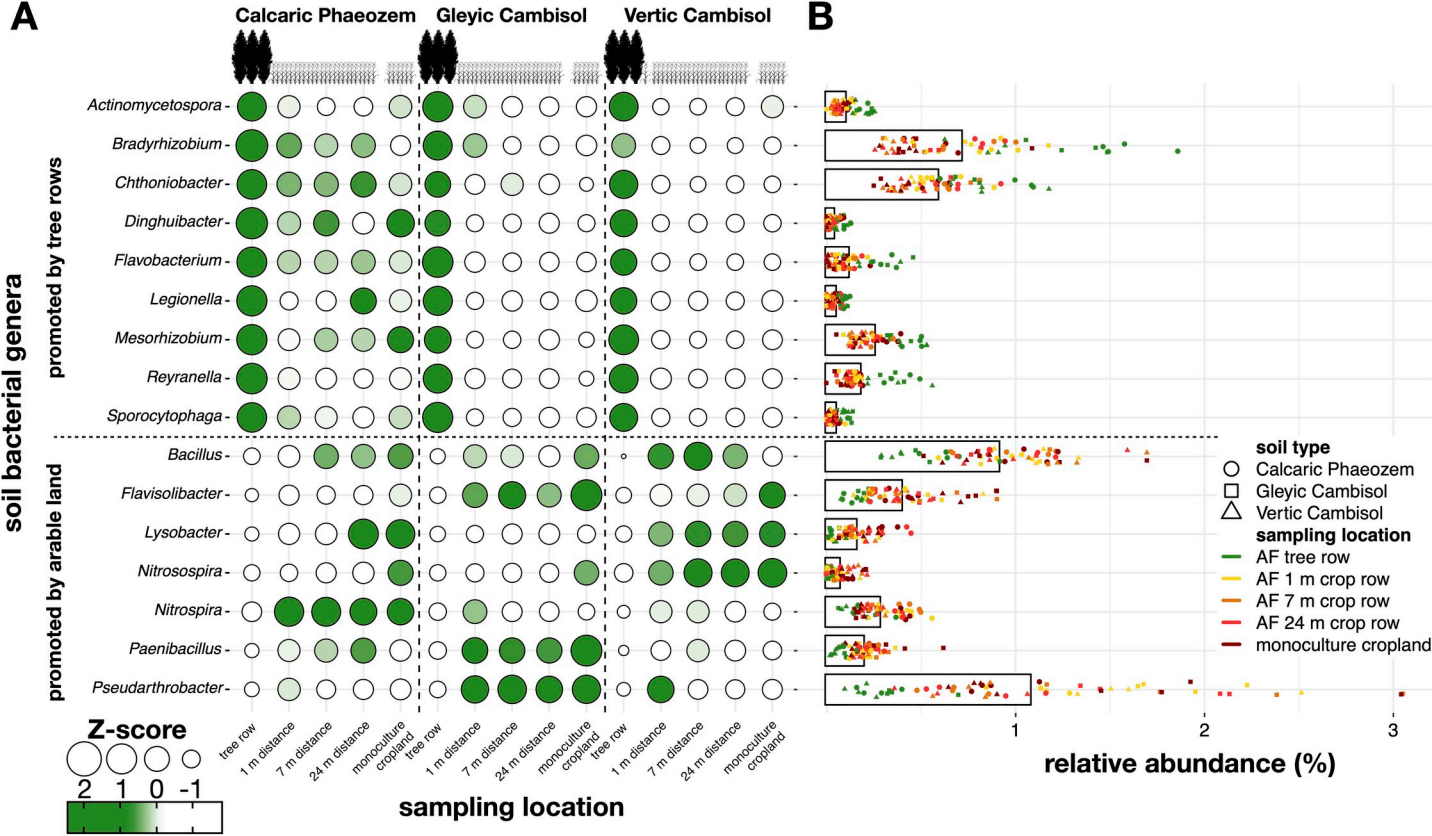
Z-score normalized relative abundance (**A**) and relative abundance (**B**) of selected taxonomic soil bacterial genera in paired temperate agroforestry and monoculture cropland systems in three different soil types. Samples in the agroforestry systems were collected in the tree row as well as at 1 m, 7 m, and 24 m distance from the tree row within the agroforestry crop row. Horizontal bars represent the mean relative abundance, coloured shapes represent individual data points (**B**).

We observed that the relative abundance of several genera of soil bacteria was enhanced by the integration of poplar rows in agricultural systems (Fig 5). For example, members of the genera *Actinomycetospora* (p ≤ 0.004), *Bradyrhizobium* (p ≤ 0.041), *Flavobacterium* (p ≤ 0.007), *Mesorhizobium* (p ≤ 0.010), *Reyranella* (p < 0.001), and *Sporocytophaga* (p ≤ 0.028) were more abundant in the tree row compared to the arable land (agroforestry crop row and the monoculture) in all three soil types. In all three soil types, the monoculture showed lower abundance of *Chthoniobacter* spp. as compared to the tree row (p ≤ 0.039). The genus *Dinghuibacter* was more abundant in the row on the Gleyic and the Vertic Cambisol soil than in the arable land (p ≤ 0.038). On the Gleyic Cambisol soil, *Legionella* affiliates showed greater relative abundance under the trees than in the agroforestry crop row and the monoculture (p ≤ 0.007).

In contrast, several groups showed a decline in their relative abundance in response to the trees (Fig 5). For example, in all three soil types, the relative abundance of *Pseudarthrobacter* affiliates was lower in the tree row than the arable land (p ≤ 0.021). On the Calcaric Phaeozem and Vertic Cambisol soil, the genus *Paenibacillus* showed lower abundance under the trees than in the arable land (p ≤ 0.015). On the Gleyic Cambisol soil, *Paenibacillus* spp. were lower in the tree row than in the monoculture (p ≤ 0.002). Likewise, lower relative abundance of *Flavisolibacter* (p ≤ 0.012) and *Nitrosospira* spp. (p ≤ 0.016) in the tree row versus the monoculture were observed in all three soil types. *Nitrospira* spp. were less abundant under the trees than in the arable land on the Calcaric Phaeozem soil (p ≤ 0.04). Additionally, *Nitrospira* spp. showed lower abundance under the trees than in the agroforestry crop row on the Vertic Cambisol soil (p ≤ 0.003). On the Calcaric Phaeozem, *Bacillus* affiliates showed lower abundance in the tree row than at 7 m and 24 m in the agroforestry crop row as well as in the monoculture (p ≤ 0.02), while on the Vertic Cambisol soil, the genus was less abundant in the tree row than in the agroforestry crop row (p ≤ 0.005).

### Abundance of N fixation gene *nifH* in soil

**Fig 6:**
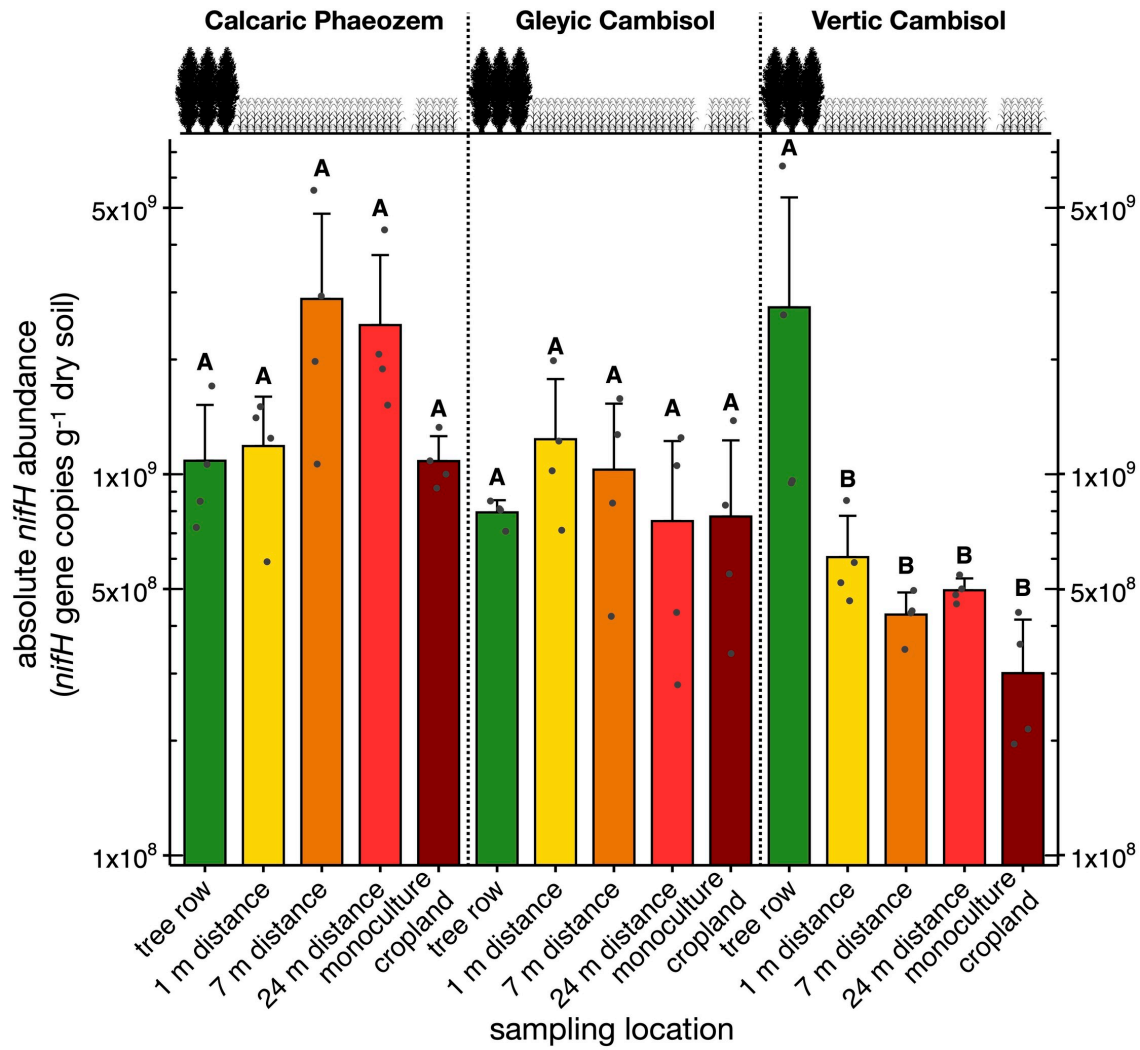
Abundance of N fixation gene *nifH* in paired temperate agroforestry and monoculture cropland systems in three different soil types. Samples in the agroforestry systems were collected in the tree row as well as at 1 m, 7 m, and 24 m distance from the tree row within the agroforestry crop row. Bars represent means with standard deviation (*n* = 4 per soil type × sampling location). Different uppercase letters indicate significant differences among the sampling locations within the same soil type (one-way analysis of variance with Tukey’s honestly significant difference test or Kruskal–Wallis test with multiple comparison extension).

The abundance of *nifH* gene on the Vertic Cambisol soil was greater in the tree row than in the crop row of the agroforestry system as well as in the monoculture system (p < 0.02) (Fig 6). In contrast, no differences in the abundance of *nifH* genes were found among sampling locations on the Calcaric Phaeozem and the Gleyic Cambisol soils.

## Discussion

### Main impacts of temperate agroforestry on soil bacterial communities

Our results revealed that poplar tree rows in temperate agroforestry increase the abundance of soil bacteria and alter the composition of the soil bacterial community, which is in line with previous findings [27, 28]. Although the integration of trees in arable land did not increase soil bacterial diversity, which agrees with the results of Banerjee *et al*. [27], tree row-associated soil bacteria enhanced the overall diversity of the system. Furthermore, the relative abundance of genera involved in soil N cycling was affected: N-fixing bacteria of the genera *Bradyrhizobium* and *Mesorhizobium* were promoted by the trees, whereas nitrifying bacteria of the genera *Nitrosospira* and *Nitrospira* were less abundant under the trees as compared to agroforestry crop row and the monoculture cropland. Overall, our study demonstrated that temperate agroforestry practice introduces a tree row-associated bacterial microbiome, which likely affects soil functional diversity and nutrient cycling.

### Abundance and alpha diversity of soil bacterial communities in temperate agroforestry systems

The greater abundance of soil bacteria under the trees on the Vertic Cambisol soil (Fig 3A) confirmed our first hypothesis agrees with our previous findings that poplar rows in temperate agroforestry systems benefit soil microorganisms [28]. Likewise, Banerjee *et al*. [27] reported greater bacterial abundance in plots with trees than without trees in Canadian agroforestry systems. Also in line with the findings of Banerjee *et al*. [27] temperate agroforestry did not affect the Shannon diversity of the soil bacterial ASVs (Fig 4A). The evenness of soil bacterial ASVs was lower in the tree rows than in the arable land of the two agroforestry systems planted with summer barley (Calcaric Phaeozem and Gleyic Cambisol soil) (Fig 4B), revealing that the tree rows harboured a greater proportion of dominant ASVs. As reported previously [28], all our agroforestry systems harboured herbaceous vegetation of high biodiversity growing below the poplar trees. Therefore, our results are consistent with the assumption that aboveground plant alpha diversity does not predict the belowground microbial alpha diversity [47]. Since the poplar trees at all our study sites originated from the same poplar clone, the response of soil bacterial diversity to agroforestry was affected by the soil type and/or management regime (crop rotation, fertilization, and soil management).

### Effect of trees in agroforestry systems on soil bacterial groups involved in soil N cycling

The relative abundance of the alpha-rhizobial genera *Bradyrhizobium* and *Mesorhizobium* were promoted by trees compared to the arable land (Fig 5). *Bradyrhizobium* and *Mesorhizobium* affiliates living in symbiosis with legumes are well-known for their capability to improve plant supplementation with N by assimilating atmospheric N_2_ [48, 49]. To further elucidate whether poplar-based agroforestry affects the genetic potential for N fixation, we quantified the *nifH* gene in soil. In a strong agreement with our previous findings [28], we observed that on the Vertic Cambisol, the tree row increased the absolute abundance of *nifH* compared to the arable land, whereas this was not found in the other two soil types (Fig 6). Although poplars are non-nodulating plants, plants found in the herbal vegetation below the trees on the Vertic Cambisol may be capable of nodulation and, thus, could contribute to N fixation. If confirmed, N fixation by herbal vegetation in the tree rows of temperate agroforestry systems would be a yet unaccounted benefit of temperate agroforestry practice.

The lower relative abundance of the nitrifiers comprising *Nitrosospira* and *Nitrospira* spp. under the trees as compared to the arable land (Fig 5) is congruent with our previous findings of reduced absolute abundance of ammonium-oxidizing bacteria in tree rows of temperate agroforestry systems as compared to the agroforestry crop rows and monoculture systems [28, 29]. In agreement with our results, the presence of trees reduced the relative abundance of *Nitrosospira* in Canadian agroforestry systems as compared to arable land [27]. The reduction of *Nitrosospira* and *Nitrospira* spp. under the trees was likely related to the absence of fertilization in the tree row [50]. Considering the increased abundance of genes involved in denitrification in the tree rows of temperate agroforestry systems [28], we conclude that agroforestry alters the genetic potential for N fixation, nitrification, and denitrification.

### Effect of agroforestry on the composition of soil bacterial community

Our results demonstrated that the composition of soil bacterial community was strongly affected by the soil type (S1 Table), which was expected considering the strong effect of soil properties on the assembly of microbial populations (e.g. [51]). Altogether, our data reveals that poplar rows in temperate cropland agroforestry system harbour a different soil bacterial community than the arable land. Furthermore, the composition of the soil bacterial community in each soil type was influenced by the sampling location within the agroforestry system (tree row, 1 m, 7 m, and 24 m distance from the tree row) or in the conventional monoculture cropland without trees (S1 Table, S2 Fig), confirming our third hypothesis. In particular, soil bacterial communities collected in the tree rows were well separated from those from arable land (agroforestry crop row and monoculture cropland) as revealed by the clustering in the NMDS (Fig 4C). Therefore, the integration of trees into crop production through agroforestry diversifies the soil microbiome by introducing tree row-associated soil bacteria that enhance the overall diversity of the system, confirming our second hypothesis. These differences on DNA-level likely reflect functional differences, as reported previously for soil microbial communities of agroforestry systems [19, 20, 52]. We do not believe, however, that the functions of bacteria in soil can be accurately predicted from partial sequences of their 16S rRNA genes, considering the poor discriminatory power of the method at species level [53]. Furthermore, biological, chemical, and physical interactions in soil [54] strongly modulate the function of the soil microbiome.

There is substantial evidence that plant communities shape the composition of soil bacterial communities [47, 55–57]. Several studies showed that this effect is mediated by plant root exudates (e.g. [58]). For example, the observation that different arable crops established different bacterial communities in their rhizosphere was accounted for plant host habitat and root exudation [55]. Secondary metabolites (syn. special metabolites) were shown to play an important role in the modulation of soil microflora by plants [59, 60]. The persistent and abundant poplar biomass and the herbaceous layer in the tree rows of our agroforestry systems [28] are expected to release large quantities of root exudates with a much higher chemical diversity than the root exudates secreted into soil by in crop plants in the agroforestry system and in conventional monoculture cropland. Surprisingly, the high diversity of the herbal vegetation under the trees and their root exudates were not associated with an increase of alpha diversity of soil bacteria (Fig 4A). We assume that the tree litter and root exudation of the trees exerted a dominant effect on soil bacterial populations due to their large biomass. Therefore, the biodiversity of herbal vegetation under the trees and its exudations did not lead to the diversification of soil bacteria.

The amount of aboveground tree litter input (leaves, twigs, and branches) typically decreases exponentially with increasing distance from the trees [61]. Previous studies showed that the assembly of soil bacterial communities was affected by the quantity [62] as well as the quality [63] of tree leaf litter. In line with our results discussed above, we assume that the accumulation, decay, and incorporation of tree litter into the soil of the tree rows were among the major effects shaping belowground microbial communities in the tree rows. Because primary decomposers of tree litter are fungi, a DNA-based inventory and comparative analysis of fungal soil community would advance our understanding of soil biology in agroforestry systems.

In addition to harboring completely different plant communities, the cropland was tilled and fertilized while these treatments were absent in the tree rows. It is well established that fertilization strongly affects bacterial community composition. Both the kind of fertilizer and its amount affect bacterial communities in soil [64]. Fertilization and tree litter provide nutrients for soil microflora. Thus, nutrient input appears to be the major factor fostering the differentiation of soil microbiome between tree rows and arable land in agroforestry systems. In addition to nutrient input, tillage has repeatedly been shown to affect belowground communities [65–67], which is plausible considering the physical impact that the tillage exerts on soil as microbial habitat [68]. Therefore, differences in nutrient input and tillage in temperate agroforestry cropland systems appear to be the major factors modulating the soil microbiome.

### Library normalization using qPCR

It is well established that the generation of amplicon sequencing libraries using PCR inevitably induces amplification bias [69], which increases with the number of PCR cycles (e.g. [70]). Therefore, is it generally recommended to limit the number of PCR cycles. Additionally, we suggest normalizing the starting quantity of target DNA prior to PCR to reduce differences in amplification among samples. For example, competition of PCR products with the primers for primer binding sites will slow down the amplification of dominant templates in samples with larger quantity of DNA faster than in samples with low amount of DNA. Because minor templates are less likely to be affected, differences in starting DNA concentration may affect relative amplification efficiencies of DNA species among samples, contributing to PCR bias. On the other hand, stochastic phenomena affecting rare templates during the first PCR cycles are likely to be stronger in samples with low starting DNA concentrations. Differences in the amount of extracted DNA are unavoidable with environmental samples and/or in studies in which a treatment affects the abundance of the target organism(s). Since our preliminary experiments showed that the quantities of target DNA varied among sampling locations, we normalized all our samples to a narrow range of below one C_q_ value using qPCR prior to library preparation (Fig 2A). Furthermore, we used a limited number of 3 touch-up cycles followed by only 25 PCR cycles for library preparation to stop the amplification before entering the plateau phase of the PCR.

## Conclusion

The composition of the bacterial communities in tree rows our temperate agroforestry systems differed from those in arable land (crop rows of the agroforestry systems and conventional monoculture croplands). Therefore, the integration of trees in arable land through agroforestry diversifies the soil microbiome by introducing and promoting tree row-associated soil bacteria that enhance the overall diversity of the system. Furthermore, we suggest that the compositional alterations induced by the tree rows result in functional diversification of the soil microbiome of agroforestry systems. Differences in plant-derived nutrients (root exudates and tree litter) and management practices (fertilization and tillage) likely contributed to the observed differences between bacterial communities of tree rows and arable land.

## Supporting information

S1 Fig Agarose gel (**A**) and calibration curve (**B**) used for densitometric quantification of library yield. The agarose gel was loaded with duplicates of a 3:1 serial dilution of a library in 0.5 × TE buffer. The agarose concentration was 1.7% (w/v) and gel electrophoresis was carried out at 4.6 V cm^-2^ for 60 min. Gels were stained in 0.1% (w/v) ethidium bromide solution for 10 min and de-stained in demineralized H_2_O for 20 min prior to visualization using UV light. For densitometry, the mean grey pixels per area unit of the library bands were determined from the agarose gel by using ImageJ version 1.52q [34].
(DOCX)

S2 Fig Venn diagram of the number of soil bacterial amplicon sequence variants (ASVs) in paired temperate agroforestry and monoculture cropland systems in three different soil types. Samples in the agroforestry systems were collected in the tree row as well as at 1 m, 7 m, and 24 m distance from the tree row within the agroforestry crop row on the Calcaric Phaeozem (**A**), Gleyic Cambisol (**B**), and Vertic Cambisol soil (**C**) (*n* = 4 per soil type × sampling location).
(DOCX)

S1 Table Permutational multivariate analysis of variance (PERMANOVA) results. PERMANOVA was performed with 999 permutations using amplicon sequence variant (ASV) count data. df = degrees of freedom; Sum Sq = sum of squares; *R*^*2*^ = coefficient of determination; *F* = pseudo-*F* ratio; *p*-values marked in bold indicate statistical significance at *p* < 0.05. ^a^ three soil types (Calcaric Phaeozem, Gleyic Cambisol, and Vertic Cambisol). ^b^ five sampling locations within each soil type, of which four were located in the agroforestry cropland (tree row, 1 m, 7 m, and 24 m distance from the tree row) and one in the adjacent monoculture cropland (Fig 1).
(DOCX)
