# Supplementary material for: Tree rows in temperate agroforestry croplands alter the composition of soil bacterial communities
Source: PLoS One. 2021 Feb 10;16(2):e0246919. doi: 10.1371/journal.pone.0246919 (PMC7875383; doi:10.1371/journal.pone.0246919)
Supplement: S1 Fig — Agarose gel (A) and calibration curve (B) used for densitometric quantification of library yield. The agarose gel was loaded with duplicates of a 3:1 serial dilution of a library in 0.5 × TE buffer. The agarose concentration was 1.7% (w/v) and gel electrophoresis was carried out at 4.6 V cm-2 for 60 min. Gels were stained in 0.1% (w/v) ethidium bromide solution for 10 min and de-stained in demineralized H2O for 20 min prior to visualization using UV light. For densitometry, the mean grey pixels per area unit of the library bands were determined from the agarose gel by using ImageJ version 1.52q [34]. (DOCX) [file pone.0246919.s001.docx]

**
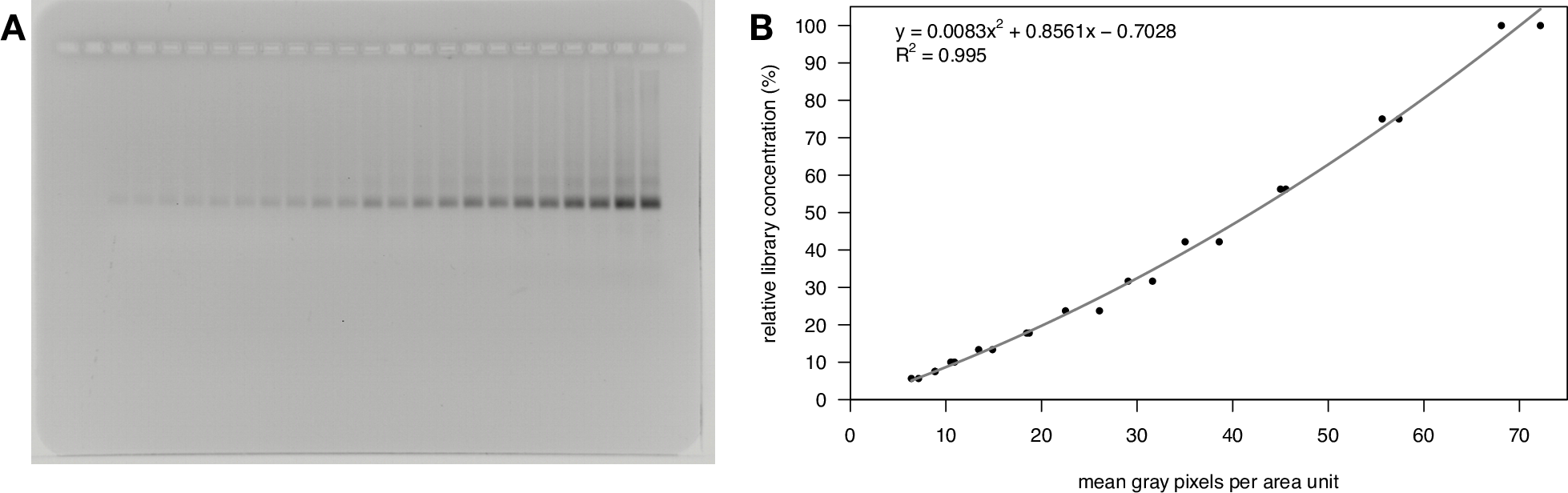
**

**S1 Fig. Agarose gel (A) and calibration curve (B) used for densitometric quantification of library yield.** The agarose gel was loaded with duplicates of a 3:1 serial dilution of a library in 0.5 × TE buffer. The agarose concentration was 1.7% (w/v) and gel electrophoresis was carried out at 4.6 V cm^-2^ for 60 min. Gels were stained in 0.1% (w/v) ethidium bromide solution for 10 min and de-stained in demineralized H_2_O for 20 min prior to visualization using UV light. For densitometry, the mean grey pixels per area unit of the library bands were determined from the agarose gel by using ImageJ version 1.52q [31].
