# Supplementary material for: Tree rows in temperate agroforestry croplands alter the composition of soil bacterial communities
Source: PLoS One. 2021 Feb 10;16(2):e0246919. doi: 10.1371/journal.pone.0246919 (PMC7875383; doi:10.1371/journal.pone.0246919)
Supplement: S2 Fig — Samples in the agroforestry systems were collected in the tree row as well as at 1 m, 7 m, and 24 m distance from the tree row within the agroforestry crop row on the Calcaric Phaeozem (A), Gleyic Cambisol (B), and Vertic Cambisol soil (C) (n = 4 per soil type × sampling location). (DOCX) [file pone.0246919.s002.docx]

**
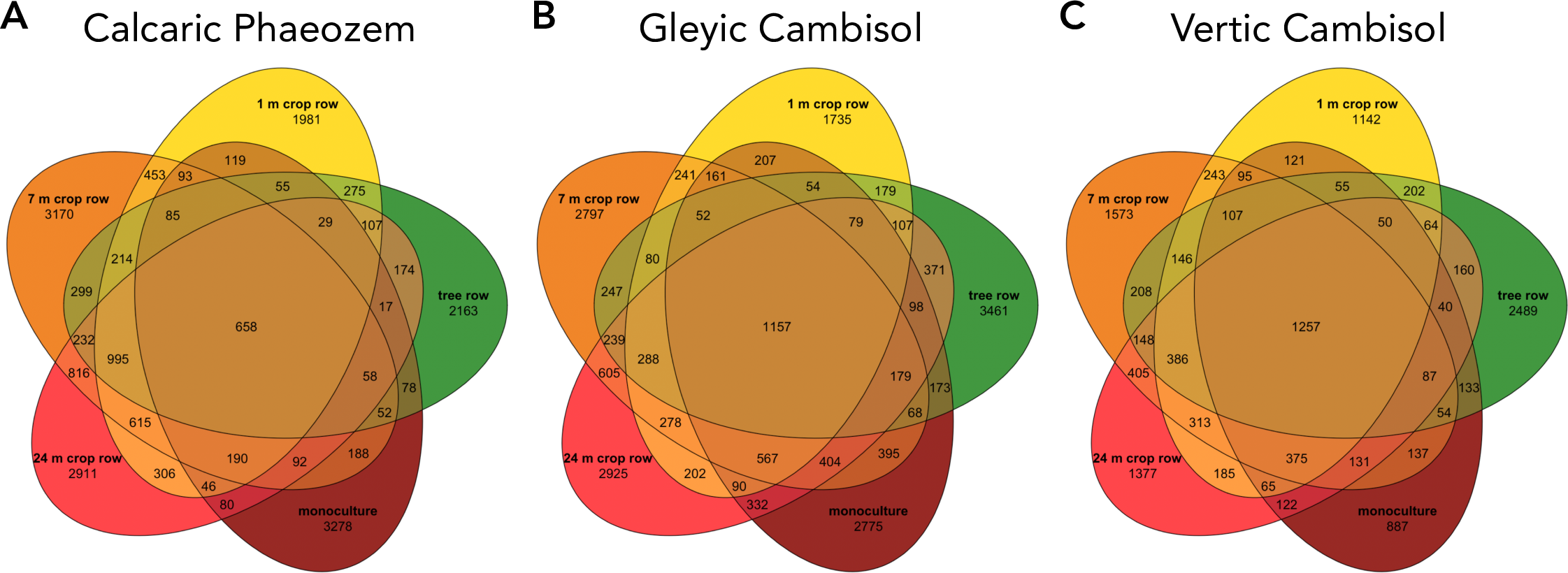
**

**S2 Fig. Venn diagram of the number of soil bacterial amplicon sequence variants (ASVs) in paired temperate agroforestry and monoculture cropland systems in three different soil types.** Samples in the agroforestry systems were collected in the tree row as well as at 1 m, 7 m, and 24 m distance from the tree row within the agroforestry crop row on the Calcaric Phaeozem (**A**), Gleyic Cambisol (**B**), and Vertic Cambisol soil (**C**) (*n* = 4 per soil type × sampling location).
