# Supplementary material for: Tree rows in temperate agroforestry croplands alter the composition of soil bacterial communities
Source: PLoS One. 2021 Feb 10;16(2):e0246919. doi: 10.1371/journal.pone.0246919 (PMC7875383; doi:10.1371/journal.pone.0246919)
Supplement: S1 Table — PERMANOVA was performed with 999 permutations using amplicon sequence variant (ASV) count data. df = degrees of freedom; Sum Sq = sum of squares; R2 = coefficient of determination; F = pseudo-F ratio; p-values marked in bold indicate statistical significance at p < 0.05. a three soil types (Calcaric Phaeozem, Gleyic Cambisol, and Vertic Cambisol). b five sampling locations within each soil type, of which four were located in the agroforestry cropland (tree row, 1 m, 7 m, and 24 m distance from the tree row) and one in the adjacent monoculture cropland (Fig 1). (DOCX) [file pone.0246919.s003.docx]

**S1 Table. Permutational multivariate analysis of variance (PERMANOVA) results.**

| **source of variance** | **df** | **Sum Sq** | ***R^2^*** | ***F*** | ***p*-value** |
| --- | --- | --- | --- | --- | --- |
| **soil type^a^** | 2 | 4.407 | 0.288 | 15.021 | **0.001** |
| **sampling location^b^** | 4 | 1.831 | 0.120 | 3.120 | **0.001** |
| **soil type^a^ × sampling location^b^** | 8 | 2.476 | 0.162 | 2.110 | **0.001** |
| **residuals** | 45 | 6.601 | 0.431 |  |  |
| **total** | 59 | 15.315 | 1.000 |  |  |

PERMANOVA was performed with 999 permutations using ASV count data. df = degrees of freedom; Sum Sq = sum of squares; *R^2^* = coefficient of determination; *F* = pseudo-*F* ratio; *p*-values marked in bold indicate statistical significance at *p* < 0.05.

^a^three soil types (Calcaric Phaeozem, Gleyic Cambisol, and Vertic Cambisol).

^b^five sampling locations within each soil type, of which four were located in the agroforestry cropland (tree row, 1 m, 7 m, and 24 m distance from the tree row) and one in the adjacent monoculture cropland (Fig 1).
